# Overcoming Heredity

**Published:** 1883-12

**Authors:** 


ARTICLE VII.

### OVERCOMING HEREDITY.

BY MRS. M. W. J.

As an illustration of what can be, and has been accomplished, through fœtal and infantile nutrition, even to the extent of *overcoming heredity*, by carefully and thoroughly following out such instructions as are asked for, for mothers, in my article in your October number, entitled "*Education of Mothers*," I desire to place on record the case of a family of five children, as narrated to me by their dentist, (who was also their father.)

First, as to their *heredity*, or, if I may be allowed the expression, their *dental antecedents*.

On the *paternal* side, the grandfather had fair average teeth, but lost them all before the age of fifty, while the grandmother lost all of hers before the age of thirty. The father, appreciating the value of his teeth, kept them in good condition by the most watchful care, but has numerous large fillings. Of his two sisters (he had no brothers), one wears an artificial denture; the other—much younger—has most of her own teeth yet, but they are very frail, and consist more of filling material than tooth-substance.

On the *maternal* side, the grandfather was toothless from the earliest recollection of his children, and the grandmother lost all of her teeth before the birth of any of the grandchildren to be mentioned. The mother wore a full upper and lower set before the conception of her first child; her oldest sister wore six upper front teeth on pivots before the age of fourteen and a full set before she was twenty; the second has very frail teeth and only retains them by the greatest care, all of them having fillings; the third has but a few ragged remnants of teeth left, and only waits for courage to have them extracted to wear a full set. No brothers.

With these facts in view, what might be expected of the teeth of the third generation, especially in a part of the country where cistern-water is used exclusively for cooking and drinking purposes, and where the soil and vegetation are most lamentably deficient in the mineral elements necessary to form sound, strong teeth.

Bearing all this in mind and having given the subject much study, the father early endeavored to impress upon his wife his views of her responsibility in the matter.

He laid before her his theories of tooth-culture by fœtal and infantile nutrition, and prescribed the diet and "drugs" by which he hoped to provide suitable nutritive elements, first to the embryo through the mother's nutrition, second to the babe through her milk, and third to the babe itself in its diet, exercise, etc.

But she responded but poorly to his efforts in the case of the first child. The prescribed diet was distasteful, with its brown bread, oatmeal porridge, etc., the lime-water and other prescriptions were unpalatable; in short, to use her own words, "other people's children had teeth, and she supposed hers would, too, and she was not going to subject herself to any such vagaries in support of mere scientific theories."

Being young and self-willed, and not long married, she had things pretty much her own way; she had the mortification of finding that her baby had soft, chalky, defective teeth, which before its third birthday had already received thirteen fillings, besides which it early suffered the loss of a lower molar, thereby, to a critical eye, marring the perfect symmetry of the features.

Concluding that it might perhaps be wiser to test the matter, radical changes were made in the diet and habits of the first child, and the mother adopted the prescribed *regime*, partially for the second child, and pretty fully for the three which followed. Bearing children rapidly, the first child being but little over four years old when the fourth was born, she was, however, unable to give that close personal attention to their teeth necessary to their absolute cleanlinsss and perfection.

Although five years elapsed between the birth of the fourth and fifth children, yet, as she was an invalid for a year previous to the birth of the last child, and for subseqent year confined to her bed for months at a time, a helpless and hopeless invalid—the children were, therefore, deprived of her personal care and attention at the most critical period of their dentition. Necessarily left much to the ministrations of ignorant and careless servants, their six year molars were neglected, while their diet, dress and exercise were often the very contrary to what they should have been, although the father, of course, gave them all the attention possible, in the little time that could be spared from his professional duties and the care of an invalid wife.

But, with all these drawbacks, let us see the results of even the partial following out of the theory of *embryonic and infantile dental nutrition:*

The oldest child had the soft, chalky *baby-teeth* so hardened and reconstructed as to require no further fillings, after the thirteen put in before the third birthday, as already stated, and now, at the age of seventeen, with the exception of a slight irregularity resulting from the unfortunate early loss of the deciduous lower molar, as stated, has a perfect set of teeth, of fine structure and quality, with only very small fissure-fillings in two of the six-year molars, which in consequence of inherited defective fissures, required attention within a few months of their eruption; all of her teeth are otherwise intact.

The second child, a boy of fifteen, has as even and sound a set of teeth as can be found anywhere; the upper cuspids only, being a little to prominent for absolute regularity.

The third, a girl of nearly fourteen, has thoroughly sound and perfect teeth, with the exception of the fissure-fillings as in the case of the first child, but is tardy in erupting the upper bicuspids.

The fourth child, with the exception of the same slight fissure-fillings, has absolutely no imperfection whatever in her teeth, either in size, color, quality or position.

There was every reason to expect very defective teeth for the last child, owing to the state of the mother's health for months preceding and years subsequent to his birth, and his consequent relegation to dry-nurses and servants.

It is too early yet to pronounce judgment upon his permanent teeth, as he is but seven years old; but as his deciduous teeth have remained intact with the exception of minute approximal fillings in the upper central incisors, which are now replaced by permanent teeth of fine quality, and as his six-year molars are of good texture, I think it may be fairly claimed that *heredity* has been overcome to a remarkable extent.

Were there but one, or even two children in this case, it might be regarded as mere *coincidence ;* but when five successive children, under the same treatment and with the same antecedents, show the same results, without a single exception, it cannot be considered in any other light than that of *cause and effect*, and the matter of embryonic and infantile nutrition becomes worthy of more serious attention than would be accorded a mere *theory* unsupported by *facts*. —*Southern Dental Journal.*

